# Oxidative Stress: Mechanistic Insights into Inherited Mitochondrial Disorders and Parkinson’s Disease

**DOI:** 10.3390/jcm6110100

**Published:** 2017-10-27

**Authors:** Mesfer Al Shahrani, Simon Heales, Iain Hargreaves, Michael Orford

**Affiliations:** 1 Neurometabolic Unit. National Hospital for Neurology and Neurosurgery, Queen Square, London WC1N 3BG, UK; mesfer.shahrani.14@ucl.ac.uk (M.A.S.); s.heales@ucl.ac.uk (S.H.); i.p.hargreaves@ljmu.ac.uk (I.H.); 2 Department of Genetics and Genomic Medicine, UCL Great Ormond Street Institute of Child Health, London WC1N 1EH, UK; 3 College of Applied Medical Sciences, King Khalid University, Abha 61481, Saudi Arabia; 4 Chemical Pathology, Great Ormond Street for Children Hospital NHS Foundation Trust, London WC1N 3JH, UK; 5 School of Pharmacy and Biomolecular Sciences, Liverpool John Moores University, Liverpool L2 2AZ, UK

**Keywords:** mitochondria, oxidative stress, reactive oxygen species, antioxidant

## Abstract

Oxidative stress arises when cellular antioxidant defences become overwhelmed by a surplus generation of reactive oxygen species (ROS). Once this occurs, many cellular biomolecules such as DNA, lipids, and proteins become susceptible to free radical-induced oxidative damage, and this may consequently lead to cellular and ultimately tissue and organ dysfunction. Mitochondria, as well as being a source of ROS, are vulnerable to oxidative stress-induced damage with a number of key biomolecules being the target of oxidative damage by free radicals, including membrane phospholipids, respiratory chain complexes, proteins, and mitochondrial DNA (mt DNA). As a result, a deficit in cellular energy status may occur along with increased electron leakage and partial reduction of oxygen. This in turn may lead to a further increase in ROS production. Oxidative damage to certain mitochondrial biomolecules has been associated with, and implicated in the pathophysiology of a number of diseases. It is the purpose of this review to discuss the impact of such oxidative stress and subsequent damage by reviewing our current knowledge of the pathophysiology of several inherited mitochondrial disorders together with our understanding of perturbations observed in the more commonly acquired neurodegenerative disorders such as Parkinson’s disease (PD). Furthermore, the potential use and feasibility of antioxidant therapies as an adjunct to lower the accumulation of damaging oxidative species and hence slow disease progression will also be discussed.

## 1. Introduction

Up to 90% of cellular metabolic energy is generated by mitochondria via the oxidative phosphorylation pathway [1]. In concert with glycolysis, the tricarboxylic acid (TCA) cycle additionally generates a small amount of energy via substrate level phosphorylation, although the vast proportion of metabolic energy is harnessed via the generation of reducing power and subsequent donation of high energy electron pairs through the electron carriers NADH and FADH2, which ultimately feed directly into mitochondrial respiratory chain (MRC). The MRC is composed of four multi-subunit proteins; complex I (NADH: ubiquinone reductase; EC 1.6.5.3), complex II (succinate: ubiquinone reductase; EC 1.3.5.1), complex III (ubiquinol: cytochrome c reductase; EC 1.10.2.2), and complex IV (cytochrome c oxidase; EC 1.9.3.1) [2], each of which contain a variety of cofactors such as hemes, flavins, and iron–sulphur clusters. In addition to these redox cofactors, two mobile electron carriers, namely coenzyme Q_10_ (ubiquinone) and cytochrome c are involved in transferring electrons between the complexes. As a result of the passage of electrons between chains, protons are pumped out of the mitochondrial matrix and into the intermembrane space, creating a proton-motive force. It is the subsequent dissipation of these protons through the mitochondrial ATPase enzyme which results in the direct phosphorylation of ADP to ATP [3].

**Figure 1:**
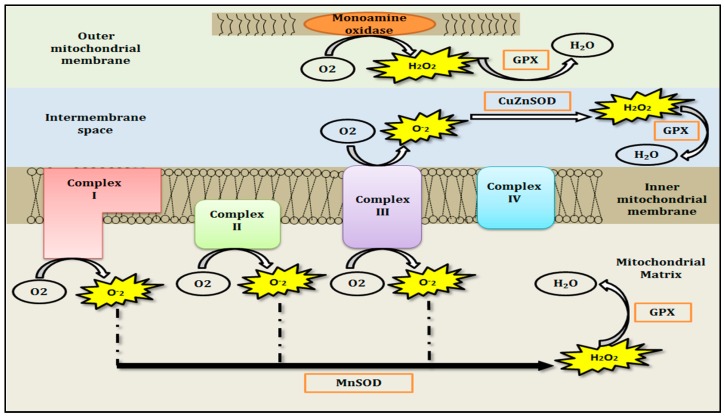
A schematic underlies the pathway of mitochondrial free radical generation and their enzymatic antioxidant defences. The mitochondrial O^•^¯_2_ is subsequently converted to H_2_O_2_ either by manganese-dependent superoxide dismutase (Mn-SOD) or copper, zinc-dependent superoxide dismutase (Cu, Zn-SOD), and then ultimately reduced to water by glutathione peroxidase (GPx).

It has been well established that the formation of reactive oxygen species (ROS) is a significant component produced during the generation of ATP. Under normal conditions, approximately 1% of total oxygen utilized by the MRC is converted to ROS, although under pathological conditions this may increase dramatically. Mitochondrial ROS, particularly in the form of the superoxide radical (O^•¯^_2_) is mostly generated either in the matrix from complex I or both in the intermembrane space and matrix from complex III [4]. Mitochondria are additionally a site of nitric oxide (NO) synthesis which in turn may form the peroxynitrite ion (ONOO^−^) when NO reacts with O^•¯^_2_, leading to the generation of equally undesirable reactive nitrogen species (RNS) [5]. O^•¯^_2_ is rapidly removed by conversion to hydrogen peroxide (H_2_O_2_) either by a manganese-dependent superoxide dismutase (Mn-SOD) or a copper, zinc-dependent superoxide dismutase (Cu, Zn-SOD), and then ultimately reduced to water by glutathione peroxidase (GPx) utilizing the active and reduced form of glutathione (GSH) as a cofactor (Figure 1) [6,7]. A large number of biomolecules have over the years been recognised as potent antioxidants. GSH itself, besides being a cofactor for the enzymatic antioxidant GPx, also serves as non-enzymatic antioxidant by directly removing free radicals as well as other oxidative agents [7]. Similarly, other powerful non-enzymatic antioxidants known to act as potent free radical scavengers include ascorbate (vitamin C) [8], *α*-tocopherol (vitamin E) [9], ubiquinol-10, the reduced form coenzyme Q_10_ [10], *α*-lipoic acid (ALA) [11], and carotenoids (*β*-carotene) [12].

It is the uncontrolled or overproduction of ROS (oxidative stress) or RNS (nitrosative stress) which can indiscriminately cause damage to cellular molecules, including DNA, proteins and lipids [13]. Furthermore, it is believed that the accumulation of these free radical species, resulting in oxidative/nitrosative stress, could lead to impaired MRC function and this in turn may be a major contributory factor to the pathophysiology of various inherited and acquired disorders [14,15].

In this review, the potential impact of oxidative stress and subsequent molecular and cellular damage will be discussed, including lessons learnt from our knowledge of the pathophysiology of a number of inherited mitochondrial disorders together with our growing understanding of perturbations observed in the more commonly acquired neurodegenerative disorders. Furthermore, we will consider the potential use of antioxidant therapies as an adjunct to standard pharmacological care as a means to limit free radical accumulation and thereby attempt to slow disease progression.

## 2. Inherited Mitochondrial Disorders

### 2.1. Inherited Mitochondrial DNA (mtDNA) Disorders

Inherited mitochondrial disorders are generally believed to be one of the most common inborn errors of metabolism, with an overall birth prevalence of about 1:5000 [3], with those resulting from mitochondrial DNA (mtDNA) mutations estimated at about 1:8000 [16]. Furthermore, mtDNA mutations are rare in children, accounting for less than 10% of all mitochondrial disorders affecting infants [17]. At least 200 pathogenic point mutations affecting the mtDNA-encoded MRC complexes I, III, and IV as well as tRNAs have recently been reported [18]. In comparison with nuclear-DNA (nDNA), mtDNA is particularly vulnerable to oxidative damage since it lacks protective histones and has limited repair mechanisms, as well as being located in close proximity to the MRC which is known to be the major source of ROS generation in the cell [19,20]. Therefore, mtDNA has a potentially higher mutation rate than nDNA. A unifying hypothesis, known as “mitochondrial catastrophe”, postulates that the accumulation of mtDNA lesions results in a decline in MRC function, which in turn, leads to the generation of further ROS, and eventually cell death [21]. This phenomenon therefore provides an insightful working hypothesis that oxidative stress could be considered as a major cause of rather than as a consequence of mtDNA disorders.

Leber’s hereditary optic neuropathy (LHON) (OMIM 540000) is one of the most well-known inherited mtDNA disorders. It is caused in most cases by three mtDNA point mutations within MRC complex I subunits [22] and results predominantly in visual loss as the main clinical feature [23]. The aetiology of oxidative stress in the mechanism of LHON disorder has been described [24,25]. It is worth emphasizing that MRC complex I is one of the major sources of ROS generation, predominately in the form of O^•¯^_2_, and it is this reactive species that is implicated to have significant effects in some, if not all of LHON disorders [26]. The inhibition of MRC complex I causes a significant increase in oxidative stress, which in turn promotes apoptosis and cell death. [27]. A recent study of patients with LHON demonstrated an increase in plasma free radical formation as well as a reduction in antioxidant levels compared to controls [28]. In addition to a reduction of MRC complex I activity and consequential increased O^•¯^_2_ levels, increases in protein carbonyl, and lipid peroxidation have also been reported in mutant mitochondrially encoded NADH dehydrogenase 6 (MT-ND6) subunit of MRC complex I. However, the mitochondrial antioxidant enzymes Mn-SOD and GPX were not altered in this study, suggesting that the mutation threshold might not be significant [29]. Interestingly, increased lipid peroxidation and raised levels of the potent oxidant hydroxyl radical (OH^•^) together with an elevation in the activity of both Mn-SOD and Cu, Zn-SOD have also been observed [30]. Consistent with this, increased levels of chemical ROS markers have been demonstrated in LHON neurons [31] as well as the marker of oxidative DNA damage, 8-hydroxy-2′-deoxyguanosine (8-OHDG), being elevated in white blood cells of LHON patients [32]. It should be further noted that in addition to endogenous ROS production, exogenous ROS sources such as those contained in tobacco smoke have also been linked to the onset of LHON disorders [33], thereby strengthening the evidence of ROS-mediated events.

### 2.2. An Inherited Mitochondrial Lipid Disorder

Barth syndrome (BTHS) (OMIM 302060), is a rare X-linked genetic disorder, characterized by cardiomyopathy, neutropenia, skeletal weakness, and growth disorders [34]. In fact, it has been previously described as a mitochondrial disorder as BTHS patients show symptoms consistent with known mitochondrial disorders [35]. It is mainly caused by a mutation in the tafazzin (*TAZ*) gene, which encodes a putative enzyme acyltransferase, an enzyme largely responsible for enzymatic remodelling of cardiolipin (CL) [36]. CL is a phospholipid component found exclusively within the inner mitochondrial membrane (IMM) and constitutes approximately 25% of the total lipid contents in mitochondria [37]. It plays a crucial role in aspects of maintaining the functional properties of mitochondrial components [38]. For example, it is required for the enhancement of the enzymatic function of the MRC complexes following CL binding [39] and its molecular interaction with all individual respiratory complexes is necessary for their assembly into super-complexes [40]. CL also plays an essential role in the retention of cytochrome *c* which protects against apoptosis [41].

The biochemical findings following MRC enzyme studies in 1983 by Barth together with other groups have indicated evidence of multiple MRC defects [42]. However, results are somewhat conflicting, suggesting the possibility that primary MRC deficiency may result in a secondary loss of other MRC activities. This latter possibility was previously been investigated in human astrocytoma cells by Hargreaves et al. in 2007 where a pharmacologically-induced MRC complex IV deficiency was found to result in a secondary loss of MRC complex II–III activity due to the progressive nature of MRC defects [43]. Interestingly, loss of CL content has been associated with mtDNA instability [44] suggesting another possible mechanism that the dysfunctional MRC encoded by mtDNA may be the consequence of oxidative damage as mtDNA structurally lacks protective histones [45]. Increased ROS levels have evidently been implicated in the *TAZ* mutation seen in cardiomyopathy which is a hallmark clinical feature of BTHS syndrome [46]. It is worth highlighting that CL is a susceptible target for oxidative damage for the following reasons: (1) CL has a naturally high unsaturated content, which is easily attacked by free radical species; (2) It is involved in the structural assembly of the MRC, a major intracellular site for ROS production; and (3) In addition to CL peroxidation, calcium-mediated detachment of cytochrome *c* from CL is induced by generating further ROS levels and this results in apoptotic cell death.

### 2.3. An Inherited Mitochondrial Protein Disorder

Friedreich ataxia (FRDA) (OMIM 229300), is a progressive neurodegenerative disorder with an autosomal recessive mode of inheritance, affecting roughly 1:50,000 live births [47]. In addition to neuronal injury in the dorsal root ganglia (DRG) and sensory peripheral nerves, FRDA patients also manifest with non-neurological symptoms including diabetes, cardiomegaly, and muscle weakness [48]. FRDA is caused by a GAA expansion in the frataxin gene, the product of which is predominantly located in mitochondria [49]. The exact role of the frataxin protein is not yet fully understood. However, it has been proposed to play crucial roles primarily in regulating iron machinery, and functioning as a mitochondrial Fe–S cluster chaperone [50,51]. In this regard, increased iron capacity and the loss of activity of mitochondrial Fe–S cluster-containing enzymes has been observed in FRDA patients, highlighting the important function of frataxin in iron metabolism [47,49,52]. In addition to its well-established role in iron metabolism, frataxin can protect against iron-mediated oxidative stress [53]. In a previous study, exposure of fibroblast obtained from patients with FRDA to ferrous ions and H_2_O_2_ reduced the viability of the cells compared to control patients [54]. The most direct evidence of the critical function of frataxin in protecting against oxidative stress however comes from the observation of a combined reduction in activity of nuclear factor E2-related factor 2 (Nrf2) and GSH levels in the YG8R mouse model of FRDA [55]. In contrast, an increased resistance to oxidative stress induced by the overexpression of mitochondrial frataxin has been reported in *Drosophila* [56]. Since the discovery of the gene in 1996, dysfunction of mitochondrial Fe–S cluster-containing enzymes including MRC complexes I and III as well as aconitase resulting in oxidative stress has been found to make a major contribution to the pathophysiology of FRDA [57].

**Figure 2:**
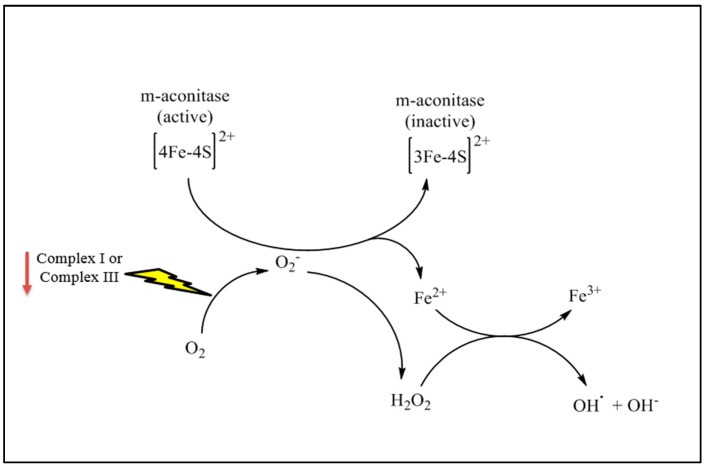
A potential mechanism for the oxidative inactivation of m-aconitase by mitochondrial O^•^¯_2_. This oxidation reaction is accompanied by the release of a ferrous ion, which subsequently contributes to the generation of OH^•^ via Fenton reaction. This scenario could consequently lead to an impairment of tricarboxylic acid (TCA) cycle capacity, a deficit in mitochondrial respiratory chain (MRC) activity, and a decline in ATP production, which in turn, leads to further oxidative damage.

Aconitase (EC 4.2.1.3) is a multi-domain enzyme, containing a closely associated iron–sulphur cluster, and exists in two slightly different structural forms: an active [4Fe-4S]^2+^ and an inactive [3Fe-4S]^1+^ cluster [58]. The active form of aconitase is highly sensitive to oxidation by the superoxide anion, which in turn, converts it to the inactive form. This oxidation reaction is accompanied by the release of a ferrous ion, which subsequently contributes to the generation of OH^•^ via the Fenton reaction [59]. As a consequence, oxidative damage to mtDNA, lipids, and proteins may occur [60]. Since the aconitase enzyme is susceptible to direct attack by free radicals, it has been recognized as an oxidative stress marker in mitochondria, suggesting it may function as a mitochondrial redox sensor [61]. Aconitase exists in two isoenzyme forms in mammalian cells: the mitochondrial aconitase (m-aconitase), and cytosolic aconitase (c-aconitase), which both enzymatically catalyse the isomerization of citrate to isocitrate. In addition to its role in the TCA cycle, c-aconitase, also known as iron-responsive protein-1 (IRP1) additionally performs a dual role in the regulation of iron homeostasis through binding to iron-responsive elements (IREs) and controlling cellular iron levels [62]. Despite the m-aconitase being identical in function (with 25% sequence homology identity) to that of c-aconitase, it is clearly not recognized to have role as an IRP [63]. However, the brain is highly dependent on m-aconitase activity [64], and is regulated by a 5′IRE in its mRNA [65]. As a consequence of inactivation of m-aconitase; neurons could be highly vulnerable to free radical attack and subsequent iron overload, resulting in a dramatic increase in oxidative stress [66]. Due to its important role in TCA cycle energy metabolism, dysfunction of aconitase may consequently lead to TCA cycle impairment, a deficit in MRC activity, and a decline in ATP production, which in turn, could lead to subsequent accumulation of ROS generation, and resultant oxidative damage (Figure 2) [67].

## 3. Parkinson’s Disease (PD)

PD is a chronic and progressive neurological disorder. It is currently ranked as the second most common neuromuscular disorder after Alzheimer’s disease, affecting roughly 1% of people almost exclusively in the over 60 age group [68]. Furthermore, the male sex is particularly susceptible to this disorder, with larger proportion of men being affected than women [69]. Clinically, PD patients commonly experience motor symptoms such as bradykinesia, tremor (particularly in the hands and/or arms), muscle stiffness (rigidity), and postural instability [70]. During later stages of the disorder, non-motor symptoms may manifest such as depression, sleep disturbances, anxiety, constipation, and in some cases dementia. Despite receiving intensive research interest over several decades and in particular with a large focus on the mechanistic aspects of PD, the exact aetiological mechanism is still poorly understood. Under normal conditions, the neurotransmitter dopamine (DA) is produced in the substantia nigra pars compacta (SNpc). However, a typical and major characteristic feature of PD is a significant depletion in its levels. The formation of intracytoplasmic eosinophilic inclusions, known as Lewy bodies (LBs), is another pathological sign of PD observed in a majority, but not all PD cases [71]. For several decades, it was postulated that PD is likely caused by environmental factors, until 1997 when the autosomal dominant mutation in the alpha-synculein (*SNKA*) gene was discovered [72]. Since this discovery, at least five other mutant genes that are linked to familial PD, including *parkin*, PTEN-induced putative kinase 1 (*PINK1*), *DJ-1*, High temperature requirement protein A2 (*HTRA2*), and leucine-rich-repeat kinase 2 (*LRRK2*) have also been identified [73]. These gene products appear to be in part localized to mitochondria and therefore may contribute towards mitochondrial dysfunction and oxidative stress [73].

**Figure 3:**
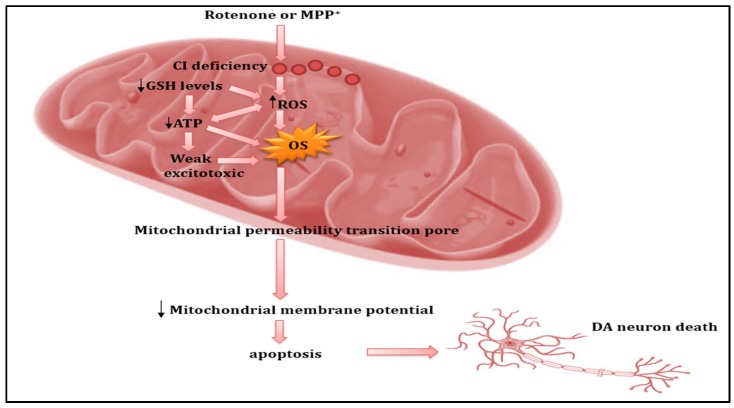
A schematic showing the role of mitochondrial dysfunction in the pathogenesis of Parkinson’s disease (PD). Neurotoxins, such as rotenone or 1-methyl-4-phenylpyridinium (MPP^+^) elicit MRC complex I deficiency, and subsequently generate reactive oxygen species (ROS), reducing levels of the antioxidant glutathione (GSH), with resulting oxidative stress. Oxidative stress induces mitochondrial permeability by transiently opening a pore, which subsequently causes depolarization of the mitochondrial membrane potential. These events ultimately lead to neural cell death via the release of pro-apoptotic mitochondrial proteins, including cytochrome *c* and apoptosis-initiating factor. DA: dopamine.

Evidence of MRC dysfunction in PD emerged in the early 1980s following the intravenous injection of 1-methyl-4-phenyl-1,2,3,4-tetrahydropyridine (MPTP) by drug abusers, producing the neurotoxin 1-methyl-4-phenylpyridinium (MPP^+^) via the monoamine oxidase-B (MAO-B) enzyme, which consequently induced Parkinson-like symptoms [72]. Similarly, in animal models, rats and primates were shown to share Parkinson-like symptoms following the administration of MPTP [74]. In parallel with MPTP, a chronic low-dose infusion of rotenone to rats additionally induced similar features of Parkinson disorders [75]. Taken together, these MRC complex I inhibitors have become widely used to create PD models to investigate the pathogenesis and therapeutic approaches for this disorder. Further studies conducted to support the role of mitochondrial dysfunctions have shown strong links to the aetiopathogenesis of PD. Studies of post-mortem PD patient brain tissue demonstrated MRC complex I deficiency in the substantia nigra and frontal cortex [76]. Consistent with these findings, MRC complex I deficiency was also shown in platelets [77] and skeletal muscle [78] from individuals with PD. In this context, it seems that a reduction of MRC complex I activity is systemic, thereby simultaneously affecting many tissues. In addition to a decrease in the activity of MRC complex I, a reduction in MRC complex III activity was further demonstrated in lymphocytes and platelets in patients with PD [79]. Remarkably, loss of MRC complex III activity may contribute to the impairment in function of MRC complex I since the stability of MRC complex I is evidently dependent on a correctly assembled MRC complex III [80]. Taken together, inhibition of MRC complex I and III can have devastating consequences, leading to excessive free radical species generation, oxidative stress and subsequent depletion of ATP levels, elevated intracellular calcium levels, excitotoxicity, and ultimately enhanced cell death (Figure 3) [81].

Any discussion would be incomplete without a reference to the interplay of iron and its contribution to mitochondrial dysfunction. Amongst all transition metals, iron is considered to be the most abundant metal in the brain, predominately in the basal ganglia [82]. It significantly contributes to the proper functioning of neurotransmitters, myelination, and mitochondria [83,84]. Brain iron metabolism is primarily regulated by transferrin and ferritin [85]. It is commonly conjugated into iron–sulphur clusters in many proteins, which have the potential ability to accept or donate electrons, particularly in the MRC pathway [86]. The evidence supporting the alteration of the iron metabolism in the neuropathology of PD is also overwhelming [87,88,89,90]. In fact, the potential mitochondrial ROS toxicity due to a defect in MRC complex I activity has been widely demonstrated in PD models [91]. Nevertheless, the exact mechanism of whether an enhanced production of ROS-induced neuronal injury is yet to be fully elucidated. Neurotoxins such the product of MPTP metabolism, commonly used to create PD models [72,75,91], have been utilized to demonstrate the potential harmful effects of the inactivation m-aconitase and high amounts of iron content on dopaminergic neurons [92]. In mice, this neurotoxin has been linked to the inactivation of m-aconitase, an increase in iron content, and a depletion of DA level [93].

**Figure 4:**
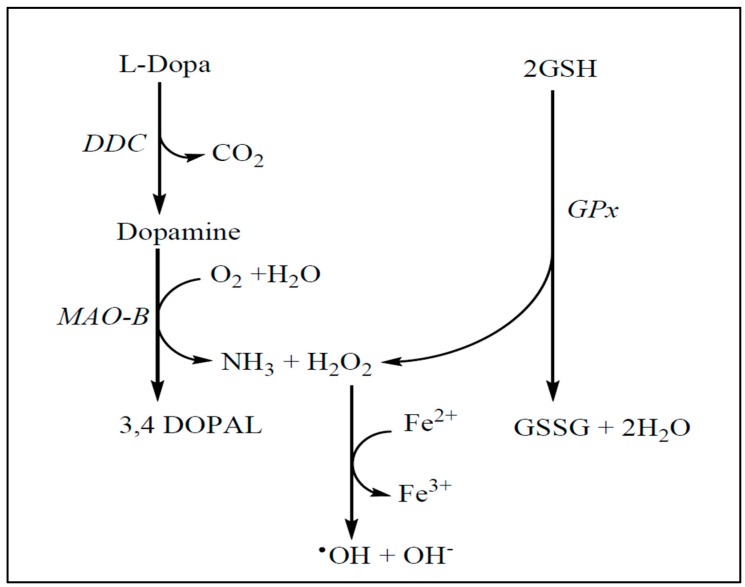
A potential mechanism of dopamine metabolism and OH^•^ radical formation in the striatum of PD patients as a consequence of iron accumulation and decline in GSH levels. DDC: dopa decarboxylase; 3,4 DOPAL: 3,4-dihydroxyphenylacetaldehyde.

It appears that excess ROS production is a common denominator of these cascades. Iron and iron derivatives contribute to the generation of the most active OH^•^ via the Fenton reaction, which in conjunction with DA autoxidation may further enhance oxidative stress, leading to degeneration of dopaminergic neurons (Figure 4) [94,95,96]. Post-mortem brain tissue from PD patients exhibited accumulation of iron content, which together with a reduction in the glutathione redox ratio of reduced glutathione/oxidized glutathione (GSH/GSSG) is a potential indicator of oxidative stress [96,97]. In the glutathione-depleted Δgsh1 cell model, on the other hand, the mitochondrial (Fe–S) cluster was unaffected, suggesting that m-aconitase is resistant to oxidative stress [98]. Furthermore, accumulation of iron was found to potentiate LB formation in the substantia nigra of PD patients, supporting the link between iron-mediated oxidative stress and the degeneration of dopaminergic neurons in PD [99].

## 4. The Role of Antioxidants in the Prevention of Oxidative Damage

Despite extensive research to elucidate the underlying mechanism of mitochondrial dysfunction in various conditions, there is no currently satisfactory treatment available. According to our recent understanding and knowledge regarding the mechanisms of mitochondrial dysfunction, it is however without doubt that mitochondrial free radical-induced oxidative damage is a plausible pathogenic facilitator in both inherited and acquired mitochondrial disorders. Alleviation of ROS/RNS free radical-mediated oxidative stress and increased availability of ATP by antioxidants could be effective therapeutic approaches to restore mitochondrial function, or at least to limit the progression of symptoms in a tremendous number of patients with mitochondrial dysfunction.

To limit free radical-induced oxidative stress, the human body is endowed with a variety of enzymatic and non-enzymatic antioxidant defence mechanisms. The two major antioxidants that protect the cell from ROS and RNS are GSH and coenzyme Q_10_ [10,100,101]. By cooperative actions, the primary function of the antioxidants is to scavenge and eliminate harmful ROS/RNS free radicals, thereby minimizing or delaying mitochondrial damage and enhancing mitochondrial bioenergetics.

### 4.1. Glutathione (GSH)

The tripeptide GSH, is a major intracellular thiol-dependent antioxidant, which protects the cellular components from free radical-induced oxidative damage [102]. Consequently, a compromised cellular GSH status results in increased production of ROS and RNS [100,101]. Despite being predominantly localised in the cytosol, GSH is also present in other intracellular organelles including, mitochondria, the nucleus, and the endoplasmic reticulum [102]. It exists in two forms: a reduced (GSH) and oxidized disulphide form (GSSG), with the ratio of reduced to oxidized forms being a key indicator of OS. In addition to its vital antioxidant role, GSH also serves as a substrate for other antioxidant defences including GPx, glutaredoxin (GRX), and thioredoxin (Trx) as well as maintaining vitamins C and E to be functionally active [7]. Accumulating evidence suggests that the depletion of GSH is associated with MRC defects [103,104]. The reduction of MRC complex I activity, followed by a depletion in GSH, has been reported previously [105]. Interestingly, results from our group have recently shown that GSH levels were significantly decreased in skeletal muscle from patients with MRC defects, compared to the control group [106]. Furthermore, patients with multiple MRC defects exhibited marked reductions in GSH levels, suggesting that oxidative stress may contribute to the pathophysiology of MRC disorders. In neurological disorders, particularly PD, it is thought that GSH depletion could be an early common event in PD pathogenesis before any significant impairment of MRC complex I and iron metabolism occur [107]. With regards to the latter however, it is uncertain whether this depletion occurs as a consequence of decreased ATP availability (required for GSH biosynthesis) or is due to increased ROS levels. Thus, the replenishment of cellular GSH could hold a promising therapeutic avenue for patients with inherited or acquired mitochondrial disorders. In rat brain, the GSH ethyl ester (GEE) derivative has been subcutaneously administered to enhance GSH levels. However, elevated brain levels were only evident post-administration directly to the left cerebral ventricle [108]. Furthermore, following co-administration with neurotoxin MPP^+^, GGE has been demonstrated to partially protect dopaminergic neuron against neurotoxicity. However, complete protection was only achieved only after pre-treating with GEE [108]. As cysteine is a major component in GSH, it hinders GSH passage across the blood–brain barrier (BBB). For this reason, the modified *N*-acetyl cysteine (NAC) form, has been effectively utilized due to it is increased ability to penetrate the BBB [109]. As such, it has also been shown to restore GSH level and consequently ameliorate free radical-induced oxidative stress [110]. Encouragingly, lesions in dopaminergic tissue have been reduced by approximately 30%, following administration with NAC, suggesting it is able to provide neuroprotection [111].

### 4.2. Coenzyme Q_10_

For many years, the clinical use of coenzyme Q_10_ or ubiquinone and its quinone analogues has been proven to be effective for treatment of mitochondrial disorders due to their capacity to augment electron transfer in the MRC, increase ATP, and enhance mitochondrial antioxidant activity, which in turn, can ameliorate the harmful effects of ROS [112]. In addition to its function as an electron carrier in the MRC pathway, Coenzyme Q_10_ also serves as a powerful free radical-scavenging antioxidant. The reduced ubiquinol form of coenzyme Q_10_ serves this function [113].

The therapeutic potential of coenzyme Q_10_ in the treatment of mitochondrial disorders took the spotlight in 1985 after Ogashara and colleagues reported sustained improvements in the clinical phenotype of patients with Kearns–Sayre syndrome (KSS) following administration with coenzyme Q_10_ [114]. More recently, Maldergem also reported that coenzyme Q_10_ therapy was beneficial to two sisters diagnosed with Leigh’s encephalopathy [115]. Remarkably, the beneficial effects of CoQ_10_ in two patients with KSS and hypoparathyroidism were also shown to help maintain calcium levels in the serum of both patients, suggesting that treatment with coenzyme Q_10_ restored the capacity of calcitriol, a hormone located in the mitochondria of proximal renal tubules [116]. Some degree of sustained improvement has been noted with some patients whose clinical features can be associated with mitochondrial disorders, such as ataxia, muscle stiffness, and exercise intolerance following implementation of coenzyme Q_10_ therapy [114]. Despite the oral coenzyme Q_10_ supplementation being significantly effective in patients with all forms of coenzyme Q_10_ deficiency, it has been shown to be only partially effective in patients who present with neurological symptoms, suggesting that these sequelae may be somewhat refractory to coenzyme Q_10_ supplementation [117]. The efficacy of the synthetic ubiquinone analogues such as idebenone has been reported in patients with mitochondrial disorders including, LOHN, FRDA, and MELAS (mitochondrial encephalomyopathy, lactic acidosis and stroke like episodes) [118,119]. It has also been recommended that patients with deficient levels of coenzyme Q_10_ should be given coenzyme Q_10_ supplementation rather than idebenone since the synthetic analogue is not a potential replacement for coenzyme Q_10_ in the MRC [120]. However, in addition to its beneficial effects, idebenone may reduce MRC complex I activity, thereby affecting the mitochondrial bioenergetics function [121]. Hence, further clinical studies regarding the overall benefits of idebenone need to be conducted to address this issue.

Both the impairment of mitochondrial function and oxidative stress have been potentially linked to neurodegenerative pathogenesis, particularly in PD. Strategies to enhance mitochondrial function and suppress oxidative stress may therefore contribute to the development of novel therapies for PD. As coenzyme Q_10_ performs two roles, one in mitochondrial energy metabolism and the other as a free-radical scavenger, low levels of coenzyme Q_10_ may therefore result in the impairment of the MRC activities as well as in the accumulation of ROS levels, and thereby contribute towards the pathogenesis of PD. Coenzyme Q_10_ deficiency associated with PD has been previously described [122,123]. A reduction in coenzyme Q_10_ level was demonstrated in the plasma [124] and platelets [125] in patients with PD, thereby suggesting that systemic effects may be important. For the first time, a UK study demonstrated that coenzyme Q_10_ levels were lower in the brain cortex of patients with PD [123]. The neuroprotective role of coenzyme Q_10_ has also been investigated in both animal and human cell models [126,127,128]. Using in vitro models of PD, coenzyme Q_10_ has been reported to protect dopaminergic neurons against neurotoxin-induced PD symptoms using either rotenone, paraquat or MPP^+^ [129]. Another study has shown that coenzyme Q_10_ treatment improved both MRC complex I and complex IV activities in skin fibroblast from PD patients [128]. To investigate the neuroprotective potential of coenzyme Q_10_ treatment in PD, 80 patients with early stage PD were randomly allocated to participate in a 16-month multicentre clinical trial [130]. Results showed that participants who received high doses of coenzyme Q_10_ had a large improvement in their motor functions, whilst lower doses only provided mild benefits. It was therefore concluded that the beneficial effect of coenzyme Q_10_ treatment may contribute to a reduction in the progression of PD.

### 4.3. Other Antioxidants

There is a considerable body of scientific literature which focuses on the beneficial effects of other antioxidants, including vitamins C and E, creatine, *α*-lipoic acid, urate, melatonin, and their derivatives as potential mediators in treating mitochondrial disorders [131,132,133,134]. Many patients with mitochondrial dysfunction, however, have not shown any significant clinical improvements when treated with theses antioxidants alone. However, it could be hypothesized that in combination, a cocktail therapy may improve mitochondrial conditions to an extent not seen previously with a single antioxidant agent. Several recent studies have also demonstrated some initial promise in the use of mitochondrial-targeted antioxidants with different modes of action to provide additional beneficial effects, such as mitoquinone (MitoQ) and mitotocopherol (MitoVitE) [135]. However, these compounds need to be further investigated to evaluate their full efficacy and safety as potential therapeutic treatments for mitochondrial disorders.

### 4.4. Ketogneic Diet (KD)

The KD, in its various forms, has successfully been used to treat patients with pharmacoresistant epilepsy [136,137,138]. Whilst the exact mechanism with regards to how the diet exerts its efficacy is not known, there is growing evidence that, in part, this may occur as result of stimulation of mitochondrial biogenesis [139]. This raises the possibility of use in patients with acquired and inherited mitochondrial disorders. Recently, we have shown that a component of the medium chain triglyceride KD, decanoic acid (C10), stimulates mitochondrial biogenesis and increases MRC complex I activity and antioxidant status in neuronal cells [140]. Furthermore, cells from patients with MRC complex I deficiency have, in some cases, been shown to respond positively to C10 exposure [141].

## 5. Conclusion Remarks

**Figure 5:**
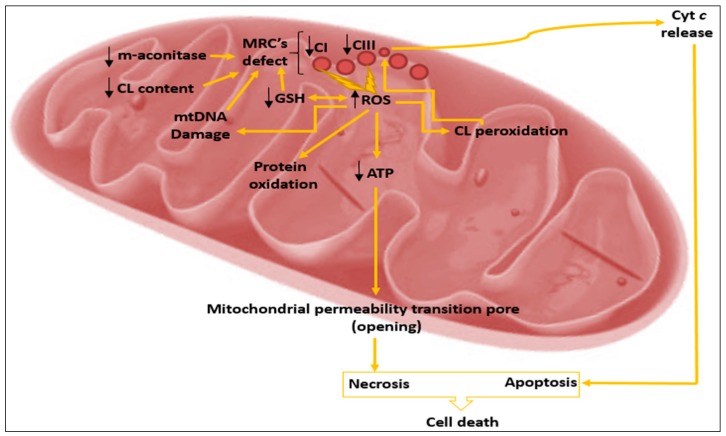
A summary of oxidative stress-induced mitochondrial damage is a common mechanistic link in the pathogenesis of inherited mitochondrial disorders and PD. CL: cardiolipin; mtDNA: mitochondrial DNA.

As highlighted in this review, free radical-induced oxidative damage to the biomolecules of the mitochondria are intrinsically linked to the pathophysiology of a number of disorders (see summary in Figure 5). Despite the number of markers available to determine evidence of oxidative stress together with its pathological consequences, few clinical centres as yet include the determination of this parameter as part of the diagnostic algorithm of patient evaluation. Furthermore, in view of the vulnerability of mitochondrial biomolecules to oxidative damage by ROS or RNS, therapeutic strategies should be targeted the free radical threshold [13] and the molecular structure targeted by these radicals. Recent advances using mitochondrial-targeted antioxidants and dietary modification may hold potential promise to provide therapeutic benefit for patients with oxidative stress-associated disorders.

## Abbreviations

ROS	Reactive oxygen species
PD	Parkinson’s disease
MRC	Mitochondrial respiratory chain
GPx	Glutathione peroxidase
GSH	Reduced glutathione
CL	Cardiolipin
mtDNA	Mitochondrial DNA
LHON	Leber’s hereditary optic neuropathy
BTHS	Barth syndrome
FRDA	Friedreich ataxia
MPTP	1-methyl-4-phenyl-1,2,3,4-tetrahydropyridine
